# Plaque‐Hepatic Targeting Nanotherapy Disrupts the PCSK9‐LOX‐1 Axis to Suppress oxLDL in Atherosclerosis

**DOI:** 10.1002/advs.77100

**Published:** 2026-08-13

**Authors:** Yi Duan, Yijie Qiu, Yan Zhu, Quan Wang, Jiangtao Lin, Yourong Duan, Qi Wang, Yi Dong

**Affiliations:** ^1^ Department of Ultrasound Xinhua Hospital School of Medicine Shanghai Jiao Tong University Shanghai China; ^2^ State Key Laboratory of Systems Medicine For Cancer, Shanghai Cancer Institute, Renji Hospital, School of Medicine Shanghai Jiao Tong University Shanghai China; ^3^ Key Laboratory for Advanced Materials and Institute of Fine Chemicals, Feringa Nobel Prize Scientist Joint Research Center, School of Chemistry and Molecular Engineering East China University of Science and Technology Shanghai China

**Keywords:** LOX‐1, PCSK9, atherosclerosis, plaque targeting, siRNA delivery

## Abstract

Atherosclerosis remains the leading cause of cardiovascular mortality, with elevated oxidized low‐density lipoprotein (oxLDL) as a key driver. oxLDL metabolism involves two critical steps: generation mediated by proprotein convertase subtilisin/kexin type 9 (PCSK9)‐induced LDLR degradation, and plaque uptake via lectin‐like oxLDL receptor‐1 (LOX‐1). Current PCSK9 inhibitors reduce oxLDL production but show limited effects on plaque oxLDL uptake and inflammation. Thus, synergistic strategies targeting both steps are urgently needed. To address this, we developed a hepatic‐plaque targeting nanoparticle, siPCSK9@PEAL NPs‐aL, based on a PEG‐PLGA‐PLL framework. The nanoparticle was surface‐functionalized with anti‐LOX‐1 antibody for plaque targeting. Concurrently, optimized particle size enabled hepatic accumulation while minimizing clearance by the reticuloendothelial system (RES), facilitating effective hepatic delivery of siPCSK9. The PLGA core allowed controlled siRNA release, and the cationic PLL layer promoted efficient condensation and protection. In vitro, this system effectively silenced PCSK9, downregulated LOX‐1, rescued mitochondrial function and reduced apoptosis. In advanced atherosclerosis mice, weekly administration significantly reduced aortic plaque burden, stabilized plaque composition, and normalized serum lipid levels. Lipidomics showed oxLDL‐associated lipid downregulation and metabolic networks remodeling. Taken together, this dual‐targeting nanodrug integrates systemic lipid‐lowering with local anti‐inflammatory effects by simultaneously inhibiting oxLDL generation and utilization, offering a promising precision therapeutic strategy for atherosclerosis.

## Graphical abstract

Self‑amplifying PCSK9–LOX‑1 feedback axis drives atherosclerotic progression by promoting oxLDL generation and endothelial uptake. A dual‑targeting nanoplatform (siPCSK9@PEAL NPs‑aL) is constructed to simultaneously silence hepatic PCSK9 for lipid lowering and block plaque LOX‑1 for anti‑inflammation. This nanotherapy disrupts the pathological cycle, reduces oxidative stress, stabilizes plaque composition, and remodels lipid metabolism, offering a promising precision strategy for atherosclerosis treatment.

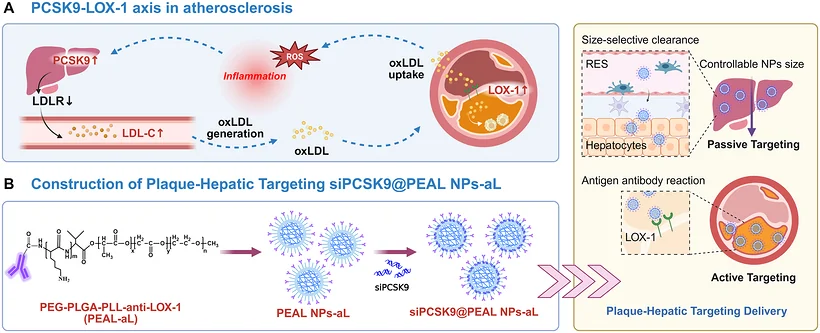

## 1 Introduction

Atherosclerosis is a chronic inflammatory disease and remains the leading cause of cardiovascular mortality worldwide [1, 2]. Elevated levels of oxidized low‐density lipoprotein (oxLDL) in the circulation represent a critical link in the initiation and progression of atherosclerosis [3, 4]. The metabolic fate of oxLDL in vivo primarily involves two key aspects: generation and uptake [5]. On one hand, proprotein convertase subtilisin/kexin type 9 (PCSK9) promotes the degradation of low‐density lipoprotein receptor (LDLR) on the surface of hepatocytes [6, 7, 8, 9], thereby reducing hepatic clearance of LDL and elevating circulating LDL‐C levels, which in turn increases the generation of oxLDL. On the other hand, in the local plaque vascular wall, oxLDL is primarily taken up via lectin‐like oxidized low‐density lipoprotein receptor‐1 (LOX‐1) expressed on the surface of endothelial cells and other cells. Binding of oxLDL to LOX‐1 activates downstream signaling pathways [10, 11, 12], leading to the release of pro‐inflammatory cytokines, enhanced oxidative stress, and endothelial dysfunction, thereby accelerating plaque formation and progression [13, 14, 15].

In recent years, therapeutic strategies targeting oxLDL generation have mainly focused on the PCSK9 pathway. Approved PCSK9 inhibitors, including the monoclonal antibodies evolocumab and alirocumab, as well as the small interfering RNA (siRNA) agent inclisiran, can significantly reduce circulating LDL‐C levels, decrease oxLDL generation, and lower the incidence of cardiovascular events [10, 15]. However, their direct intervention on local oxLDL uptake in the vascular wall and oxidative stress within plaques remains relatively limited. In contrast, drug development targeting oxLDL uptake is still largely confined to preclinical stages. Limited studies have shown that LOX‐1 inhibition can reduce oxLDL uptake, attenuate inflammation and oxidative stress, thereby improve the prognosis of atherosclerosis [16, 17]. Nevertheless, when circulating LDL‐C levels remain elevated, the persistent generation of oxLDL continues to drive disease progression. Therefore, in the current landscape of atherosclerosis treatment, combination therapeutic strategies that synergistically inhibit both the generation and utilization of oxLDL hold significant clinical necessity and potential value.

Accordingly, this study proposes a novel therapeutic strategy for atherosclerosis that simultaneously targets PCSK9/LDLR‐mediated oxLDL generation and LOX‐1‐mediated local oxLDL uptake, thereby synergistically blocking the pathological metabolism of oxLDL at both the systemic and plaque‐local levels (Scheme 1A). To this end, we developed a plaque‐hepatic dual‐targeting engineered nanoparticle, designated as siPCSK9@PEAL NPs‐aL. This nanoparticle is constructed with a polyethylene glycol‐poly(lactic‐co‐glycolic acid)‐poly‐L‐lysine (PEG‐PLGA‐PLL, PEAL) framework, surface‐functionalized with anti‐LOX‐1 monoclonal antibody (anti‐LOX‐1 mAb) and electrostatically loaded with siPCSK9. The biodegradable PLGA core enables controlled release of siRNA [18], while the cationic PLL layer facilitates efficient siRNA condensation, protects it from degradation, and promotes endosomal escape [19, 20]. Through surface modification with anti‐LOX‐1 mAb, the nanoparticles actively target LOX‐1‐overexpressing endothelial cells within atherosclerotic plaques, thereby blocking local oxLDL uptake and mitigating oxidative stress. Concurrently, by optimizing particle size, the nanoparticles achieve passive accumulation in the liver while bypassing rapid clearance by the reticuloendothelial system (RES), enabling effective delivery of siPCSK9 to hepatocytes. This leads to PCSK9 gene silencing and subsequent upregulation of LDLR, which reduces circulating native LDL levels and decreases oxLDL generation (Scheme 1B).

**SCHEME 1:**
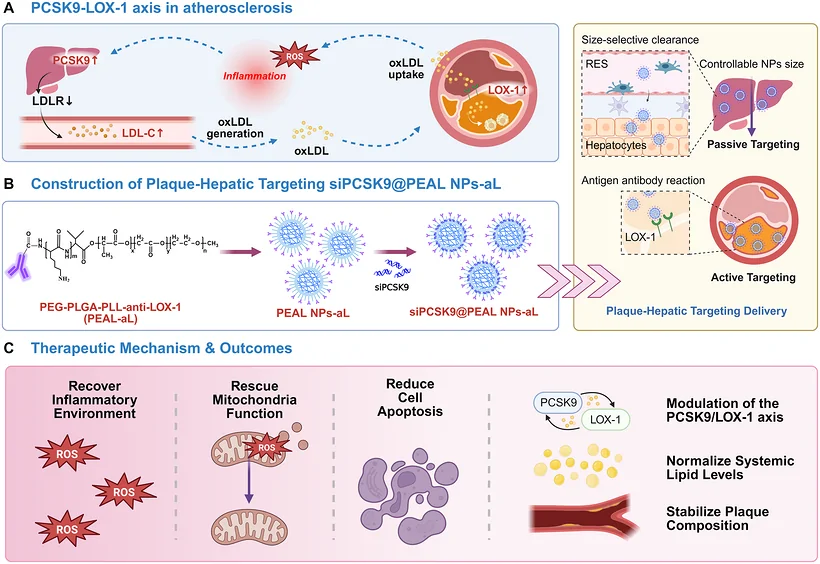
Plaque‐hepatic targeting nanotherapy disrupts oxLDL generation and uptake for atherosclerosis therapy. (A) PCSK9 induces LDLR degradation to increase oxLDL generation, while LOX‐1 mediates oxLDL uptake in plaque endothelialocytes, forming a self‐reinforcing pathological cycle. (B) Construction and dual‐targeting delivery pathway of siPCSK9@PEAL NPs‐aL. (C) Therapeutic outcomes including PCSK9/LOX‐1 axis inhibition, mitochondrial function recovery, inflammation reduction, and plaque stabilization.

Functionally, the siPCSK9@PEAL NPs‐aL effectively silenced PCSK9 gene expression, downregulated LOX‐1 expression, rescued mitochondrial membrane potential and respiratory function, inhibited compensatory glycolysis, reduced cell apoptosis, and promoted cell proliferation. In the ApoE^−^/^−^ mouse model of advanced atherosclerosis, weekly intravenous administration significantly reduced aortic plaque burden, attenuated plaque fibrosis, stabilized plaque composition, normalized serum LDL‐C, triglyceride (TG), and total cholesterol (T‐CHO) levels, and alleviated hepatic lipid accumulation. Lipidomics analysis further revealed selective downregulation of oxLDL‐associated pro‐atherogenic lipids and remodeling of the associated lipid metabolic networks (Scheme 1C). Collectively, by integrating liver targeting with plaque targeting, this study designed a precise dual‐pathway inhibition strategy against oxLDL in the context of atherosclerosis treatment. This dual‐targeted nanodrug delivery system is expected to achieve synergistic effects of systemic lipid‐lowering and local anti‐inflammatory actions, providing new insights and potential strategies for the precision therapy of atherosclerosis.

## 2 Results and Discussion

### 2.1 PCSK9‐LOX‐1 Axis Regulated oxLDL in Atherosclerosis

To validate the vital role of PCSK9/LOX‐1 proteins in atherosclerosis pathogenesis, we screened lipid metabolism‐related proteins in aortic tissues from three established atherosclerotic mouse models [21, 22]: high‐fat diet (HFD)‐fed wild‐type mice, ApoE^−^/^−^ mice, and LDLR^−^/^−^ mice (Figure 1A and Figure S1). Western blotting confirmed significant downregulation of ApoE and LDLR in their respective models, validating the reliability of these systems for studying atherosclerosis, while a faint expression was occasionally observed in knockout mice due to non‐specific antibody binding. (Figure 1B). Among the screened proteins, LOX‐1 was markedly upregulated in the aortic arches across all three models, whereas CD36 expression remained largely unchanged. PCSK9 was also consistently elevated in all groups, particularly in LDLR^−^/^−^ mice due to impaired LDLR feedback regulation (Figure 1B). Moderate upregulation of CD163 and CD206 was observed but likely reflects macrophage infiltration rather than a primary pathogenic driver.

**FIGURE 1:**
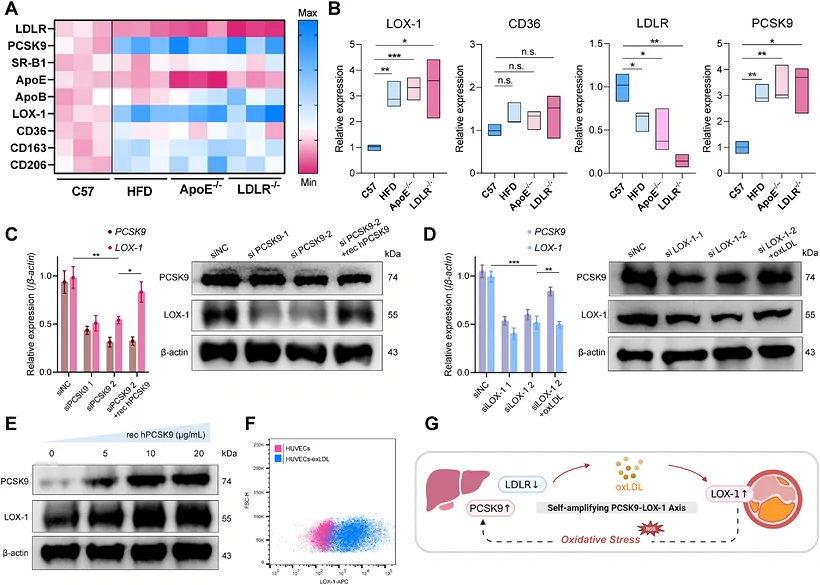
PCSK9‐LOX‐1 axis in atherosclerosis. (A) Western blot screening of key lipid metabolism‐related proteins in aortic tissues from HFD‐fed wild‐type, ApoE^−^/^−^, and LDLR^−^/^−^ mice models of atherosclerosis. (B) Quantitative analysis of different protein expression levels across the three models, normalized to β‐actin (*n* = 3). (C,D) qRT‐PCR and Western blot showing LOX‐1/PCSK9 mRNA and protein levels following knockdown with two independent siRNAs in oxLDL‐pretreated HUVECs, and rescue with rhPCSK9 or oxLDL re‐stimulation. (E) Dose‐dependent upregulation of LOX‐1 protein in oxLDL‐pretreated HUVECs treated with rhPCSK9. (F) Flow cytometry analysis of surface LOX‐1 expression on HUVECs with or without oxLDL pretreatment. (G) Schematic illustration of the self‐amplifying PCSK9‐LOX‐1 positive feedback axis in endothelial cells under atherogenic conditions. Data are presented as mean ± SD unless otherwise indicated. *p < 0.05, **p < 0.01, ***p < 0.001, n.s. no significance.

Emerging evidence implicates that PCSK9 and LOX‐1 form a self‐amplifying positive feedback axis central to atherosclerosis pathogenesis [10, 11, 12]. This crosstalk links systemic hyperlipidemia to localized plaque pathology in vascular tissues under inflammatory conditions, where PCSK9 and LOX‐1 mutually impact on each other's expression. Given the robust and concordant upregulation of PCSK9 and LOX‐1, we validated their mutual regulation in vitro using oxLDL‐pretreated HUVECs as a model of the atherogenic endothelium [23]. Knockdown of PCSK9 with two independent siRNAs significantly reduced LOX‐1 mRNA and protein levels, an effect reversed by supplementation with recombinant human PCSK9 (rhPCSK9, 10 µg mL^−1^) (Figure 1C). Reciprocally, LOX‐1 knockdown decreased PCSK9 expression, which was restored by oxLDL re‐stimulation (Figure 1D). Furthermore, treatment with increasing rhPCSK9 concentrations (5–20 µg mL^−1^) induced dose‐dependent LOX‐1 upregulation (Figure 1E). These bidirectional loss‐ and gain‐of‐function experiments confirm a positive feedback relationship between PCSK9 and LOX‐1 in endothelial cells under pro‐atherogenic conditions.

Flow cytometry further demonstrated significant surface LOX‐1 upregulation on oxLDL‐pretreated HUVECs compared to untreated controls (Figure 1F), supporting its accessibility and inducibility in the atherosclerotic microenvironment. These findings establish the PCSK9‐LOX‐1 axis as a self‐amplifying driver linking systemic lipid dysregulation to localized plaque inflammation and oxidative stress (Figure 1G), consistent with prior reports of mutual regulation in vascular tissues [10, 24, 25].

### 2.2 Construction of the Plaque‐Hepatic Targeting Nanoparticle

Building on these mechanistic insights, we engineered an anti‐LOX‐1 monoclonal antibody (aL)‐conjugated PEGylated PLGA‐PLL nanoparticle platform loaded with siPCSK9 (siPCSK9@PEAL NPs‐aL). The PEG segment provides stealth properties and prolonged circulation, the biodegradable PLGA core enables controlled siRNA release, and cationic PLL facilitates efficient encapsulation, protection from nuclease degradation, and endosomal escape. Anti‐LOX‐1 conjugation was achieved via EDC/NHS‐mediated amide coupling (Figure S2) [26]. The nanoparticles were synthesized by double‐emulsion combined with electrostatic adsorption. Transmission electron microscopy (TEM) revealed uniform spherical morphology with a well‐defined core‐shell bilayer structure, approximately 100 nm in diameter (Figure 2A). Dynamic light scattering (DLS) confirmed hydrodynamic diameters of 100.33 ± 2.68 nm (PDI = 0.174) for bare PEAL NPs, 111.60 ± 3.20 nm (PDI = 0.189) for PEAL NPs‐aL, and 119.19 ± 2.12 nm (PDI = 0.186) for siPCSK9@PEAL NPs‐aL (Figure 2B). The modest size increases upon conjugation and loading indicates successful surface functionalization with minimal aggregation. Zeta potential measurements showed +15.80 ± 0.56 mV for bare PEAL NPs, +13.92 ± 0.77 mV for PEAL NPs‐aL, and +5.27 ± 0.60 mV for siPCSK9@PEAL NPs‐aL (Figure 2C), confirming effective electrostatic adsorption of negatively charged siRNA onto the cationic nanoparticle surface. Fourier‐transform infrared (FTIR) spectroscopy tracked functional group changes (Figure 2D). The pristine PEAL polymer exhibited characteristic O–H (3437 cm^−1^), C–H (2895 cm^−1^), methyl/methylene (1200–1400 cm^−1^), and C–O–C (1105 cm^−1^) peaks. Carboxylation introduced a new C = O peak at 1628 cm^−1^. Antibody conjugation produced amide I (∼1634 cm^−1^) and amide II (1584 cm^−1^) bands, along with enhanced intensity at 1459 cm^−1^ (C = N from antibody imidazole rings), verifying successful covalent linkage.

**FIGURE 2:**
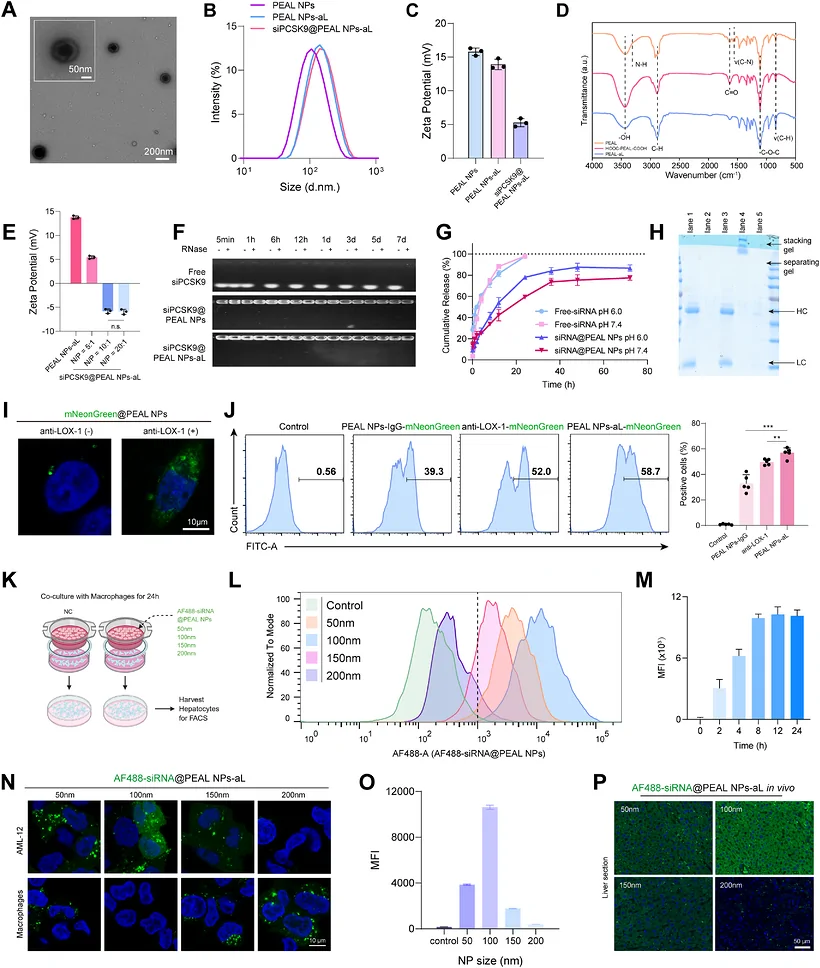
Characterization of siPCSK9@PEAL NPs‐aL. (A) Representative TEM images of siPCSK9@PEAL NPs‐aL. (B,C) Hydrodynamic size distribution and Zeta potential by DLS for bare PEAL NPs, PEAL NPs‐aL, and siPCSK9@PEAL NPs‐aL. (D) FTIR spectra of pristine PEAL polymer, carboxylated PEAL, and antibody‐conjugated PEAL‐aL. (E) Zeta potential measurements at different N/P ratios. (F) Agarose gel electrophoresis after RNase A incubation (0.1 µg mL^−1^, 37°C) at multiple time points (5 min to 3 d). (G) In vitro release profiles of siRNA from siPCSK9@PEAL NPs‐aL at pH 7.4 and pH 6.0. (H) SDS‐PAGE with Coomassie Brilliant Blue staining under various conditions: lane 1, free anti‐LOX‐1 antibody; lane 2, bare PEAL NPs; lane 3, PEAL NPs‐aL; lane 4, mixture of PEAL NPs + free antibody; lane 5, dialysate. (I) CLSM images of oxLDL‐pretreated HUVECs incubated with mNeonGreen‐loaded PEAL NPs (aL‐) versus PEAL NPs‐aL (aL+) (scale bar = 10 µm). (J) Flow cytometry quantification of fluorescent‐positive cells in oxLDL‐pretreated HUVECs after treatment with control, PEAL NPs‐IgG‐mNeonGreen, aL‐mNeonGreen, and PEAL NPs‐aL‐mNeonGreen. (K) Schematic of the Transwell co‐culture system mimicking the liver RES microenvironment. (L) Flow cytometry qualification of hepatocyte uptake of AF488‐siRNA@PEAL NPs of varying sizes (50 nm, 100 nm, 150 nm, and 200 nm). (M) Time‐course flow cytometry analysis of nanoparticle uptake in hepatocytes. (N) CLSM images of co‐cultured AML‐12 hepatocytes and macrophages after 24 h incubation with AF488‐siRNA@PEAL NPs of different sizes (scale bar = 10 µm). (O) Flow cytometry quantification of hepatocyte uptake of AF488‐siRNA@PEAL NPs of varying sizes (50 nm, 100 nm, 150 nm, and 200 nm). (P) Fluorescence microscopy of liver sections from C57 mice 24 h post tail‐vein injection of siPCSK9@PEAL NPs‐aL of different sizes (scale bar = 10 µm). Data are presented as mean ± SD unless otherwise indicated. *p < 0.05, **p < 0.01, ***p < 0.001, n.s. no significance.

Following successful synthesis of the nanoparticles, we next optimized siPCSK9 encapsulation into PEAL NPs‐aL at different nitrogen‐to‐phosphate (N/P) ratios, where N represents cationic amine groups from PLL and P represents phosphate groups from siRNA [27]. At N/p = 5:1, the nanoparticles retained a positive surface charge of +5.43 ± 0.31 mV. At N/p = 10:1, the zeta potential reversed to −5.80 ± 0.46 mV, reflecting near‐complete charge neutralization and effective siRNA condensation. Further increasing to N/p = 20:1 resulted in a slightly changed potential, suggesting that additional cationic groups provide no meaningful improvement in siRNA loading (Figure 2E). We selected N/p = 10:1 as the optimal ratio for all subsequent synthesis of siPCSK9@PEAL NPs‐aL. This ratio achieved near‐complete charge neutralization while maintaining favorable physicochemical properties for targeted delivery. RNase A challenge assay is conducted to assess the protective capacity of PEAL NPs‐aL against nuclease degradation (Figure 2F). Free siPCSK9 showed rapid degradation, with complete loss of the intact band within 1 h of RNase A exposure and no detectable RNA remaining thereafter. In contrast, siRNA encapsulated in both siPCSK9@PEAL NPs and siPCSK9@PEAL NPs‐aL remained largely intact even after 3 days of continuous RNase A treatment. The majority of the siRNA band persisted at its original position, with only faint low‐molecular‐weight fragments observed, indicating substantial protection by the nanoparticle formulation. These findings demonstrate the synthesized nanoparticles can effectively shields siPCSK9 from RNase‐mediated degradation, due to tight electrostatic condensation within the cationic PLL layer and steric hindrance provided by the PEG corona.

Then, we evaluated the pH‐responsive release behavior of siPCSK9 from PEAL NPs‐aL in PBS at pH 7.4 and pH 6.0‐microenvironment of atherosclerotic plaques (Figure 2G). At pH 7.4, siRNA release was gradual, reaching a cumulative release of 73.63% at 36 h with a slower kinetic profile. In contrast, under acidic conditions (pH 6.0), release accelerated significantly, achieving a cumulative release of 77.98% by approximately 24 h. The faster and more complete release at pH 6.0 is consistent with protonation of the cationic PLL layer. These results indicate that siPCSK9@PEAL NPs‐aL exhibit pH‐sensitive release properties, enabling rapid and efficient siRNA liberation in the acidic inflammatory environment of atherosclerotic plaques.

### 2.3 Plaque‐Hepatic Targeting Efficiency In Vitro and In Vivo

SDS‐PAGE with Coomassie Brilliant Blue staining under reducing conditions confirmed covalent conjugation (Figure 2H). Free anti‐LOX‐1 antibody displayed heavy (55 kDa) and light (25 kDa) chain bands. Bare PEAL NPs showed no protein bands. Antibody‐conjugated PEAL NPs‐aL exhibited near‐complete absence of free chains, with residual staining in the stacking gel region. Physical mixture retained prominent free antibody bands, while dialysate showed markedly reduced bands, indicating high conjugation yield and efficient removal of unbound antibody by dialysis.

To assess targeting efficiency, mNeonGreen‐loaded PEAL NPs (aL+ and aL‐) were incubated with oxLDL‐pretreated HUVECs. Confocal laser scanning microscopy (CLSM) showed markedly enhanced intracellular green fluorescence in the aL+ group, with intense and uniform cytoplasmic distribution indicative of receptor‐mediated internalization, compared to weak, sporadic fluorescence in the aL‐ group (Figure 2I). Flow cytometry quantified these differences (Figure 2J). Control cells exhibited negligible fluorescence (0.56% positive). Non‐targeted PEAL NPs‐IgG‐mNeonGreen (isotype control) yielded 39.3% positive cells. Free anti‐LOX‐1 antibody loaded with mNeonGreen achieved 52.0% positive cells. The targeted PEAL NPs‐aL‐mNeonGreen group reached the highest proportion (58.7%), significantly outperforming the isotype control (*p* < 0.001) and free antibody group (*p* < 0.01). These results demonstrated anti‐LOX‐1 conjugation substantially enhances nanoparticle uptake in LOX‐1‐overexpressing endothelial cells, providing strong proof‐of‐concept for the active plaque‐targeting capability of siPCSK9@PEAL NPs‐aL.

Given the extensive prior evidence that the liver reticuloendothelial system (RES) — preferentially allows nanoparticles smaller than ∼150 nm to pass into the Disse space for hepatocyte uptake [28, 29], we next investigated the impact of nanoparticle size on hepatic targeting, co‐cultured hepatocytes and macrophages in a Transwell system to mimic the liver RES microenvironment in vivo (Figure 2K). Nanoparticles loaded with AF488‐labeled siRNA (AF488‐siRNA@PEAL NPs) of different sizes were applied to macrophages on the upper chamber for 24 h. Hepatocytes were then collected and analyzed by flow cytometry for mean fluorescence intensity (MFI) (Figure 2L). Nanoparticles of 100 nm exhibited the highest hepatic uptake (MFI = 10,621.7), significantly outperforming other sizes. In contrast, 200 nm nanoparticles showed uptake nearly equivalent to the negative control (MFI = 388.0), while 50 nm (MFI = 3853.7) and 150 nm (MFI = 1763.0) displayed weaker uptake compared to the 100 nm formulation. Confirming the optimized dual‐targeted nanoparticles can effectively bypass the RES to effectively deliver payload to hepatocytes. Time‐course analysis of AF488‐siRNA@PEAL NPs uptake in hepatocytes by flow cytometry (Figure 2M) revealed a time‐dependent pattern. Fluorescence intensity gradually increased from 2 to 4 h, reaching a peak at 8–10 h, consistent with progressive internalization and accumulation in the target cells.

In parallel, we performed CLSM on co‐cultured AML‐12 hepatocytes and macrophages after 24 h incubation with AF488‐labeled nanoparticles of different sizes (Figure 2N). In hepatocytes, 100 nm nanoparticles displayed the strongest green fluorescence, indicating superior uptake, whereas 200 nm nanoparticles showed almost no detectable signal. In macrophages, the pattern was reversed: 200 nm nanoparticles exhibited the most prominent AF488 fluorescence, reflecting preferential RES clearance by larger particles, while the 100 nm group showed the weakest macrophage uptake.

Following in vitro validation, C57 mice liver sections were collected 24 h post AF488@PEAL NPs‐aL‐injection were examined for fluorescence distribution (Figure 2O). Fluorescence exhibited a punctate pattern in parenchymal hepatocytes, with the 100 nm formulation showing markedly higher intensity compared to the 200 nm group, which displayed extremely weak signal. These observations underscore the size‐selective advantage of ∼100 nm nanoparticles for efficient hepatocyte targeting with minimal macrophage sequestration. Then, we performed live fluorescence imaging in ApoE^−^/^−^ mice with advanced atherosclerosis using DiR@PEAL NPs‐aL tail‐vein injection. Abdominal aorta imaging showed rapid but transient accumulation of free DiR (Figure 3A and Figure S3), with peak fluorescence at 8 h, nearly undetectable at 48 h. In contrast, DiR@PEAL NPs‐aL exhibited progressive plaque enrichment, with significant fluorescence detectable at 8 h, further increasing at 24 and 48 h, indicating sustained lesion retention. Ex vivo organ imaging at revealed that both free DiR and DiR@PEAL NPs‐aL accumulated predominantly in the liver and lungs (Figure 3B,C). However, DiR@PEAL NPs‐aL showed significantly higher liver fluorescence intensity and markedly lower lung accumulation, consistent with size‐dependent passive hepatic targeting and reduced pulmonary clearance.

**FIGURE 3:**
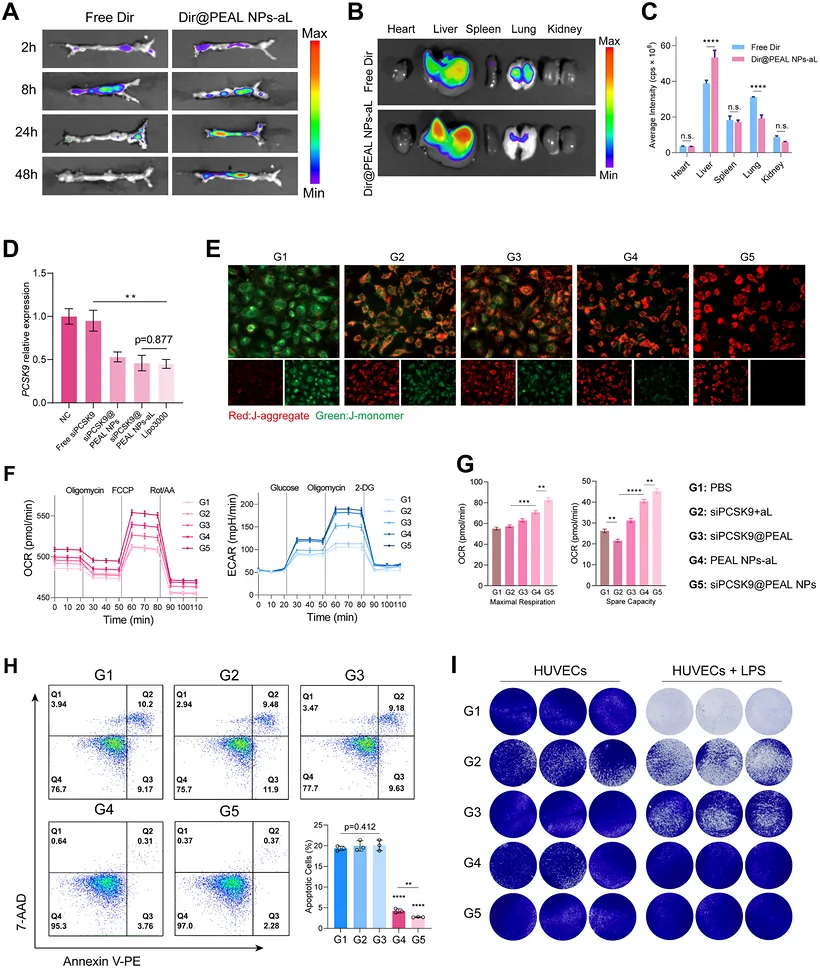
Intracellular Therapy of siPCSK9@PEAL NPs‐aL. (A) Representative images of ex vivo fluorescence imaging of the abdominal aorta at 2 h, 8 h, 24 h, and 48 h post tail‐vein injection of free DiR or DiR@PEAL NPs‐aL. (B) Representative images of ex vivo fluorescence imaging of main organs at 48 h post tail‐vein injection of free DiR or DiR@PEAL NPs‐aL. (C) Corresponding quantification results (*n* = 3). (D) qRT‐PCR analysis of PCSK9 mRNA levels in oxLDL‐pretreated HUVECs after 48 h treatment with different siPCSK9 formulations. (E) JC‐1 staining for mitochondrial membrane potential in oxLDL‐pretreated HUVECs after 24 h treatment (scale bar = 50 µm). (F) Seahorse XF analysis of OCR and ECAR in oxLDL‐pretreated HUVECs after 24 h treatment. (G) Maximal respiration and space capacity quantification from Seahorse extracellular flux analysis (*n* = 3). (H) Flow cytometry analysis of apoptosis (Annexin V+/7‐AAD+) in oxLDL‐pretreated HUVECs after 48 h treatment. (I) Colony formation assays in untreated and oxLDL‐pretreated HUVECs after 48 h, demonstrating enhanced proliferation recovery in targeted and LOX‐1 blockade groups. Data are presented as mean ± SD. *p < 0.05, **p < 0.01, ***p < 0.001, ****p < 0.0001 (one‐way ANOVA with Tukey's post hoc test; n.s. = not significant).

These results provide direct evidence of active plaque targeting mediated by anti‐LOX‐1 conjugation, combined with favorable biodistribution characterized by efficient hepatic enrichment. The prolonged plaque retention and preferential liver uptake support the dual plaque‐hepatic targeting mechanism of siPCSK9@PEAL NPs‐aL, contributing to its therapeutic efficacy in disrupting the PCSK9‐LOX‐1 axis while minimizing off‐target exposure (Figure S4).

### 2.4 PCSK9‐LOX‐1 Axis Inhibition and Intracellular Therapy of siPCSK9@PEAL NPs‐aL

Beyond validated plaque‐hepatic targeting ability, we then aim to study intracellular impact of siPCSK9@PEAL NPs‐aL. Gene silencing efficiency of siPCSK9 delivered was measured in oxLDL‐pretreated HUVECs by qRT‐PCR after different treatments (Figure 3D). The NC group exhibited baseline PCSK9 expression, while free siPCSK9 resulted in only modest knockdown. Non‐targeted siPCSK9@PEAL NPs achieved moderate silencing (46.91%). Notably, the siPCSK9@PEAL NPs‐aL group demonstrated the most potent knockdown, reducing PCSK9 mRNA levels by 59.98% compared to other groups. As a positive control, commercial Lipofectamine 3000‐mediated transfection of siPCSK9 yielded nearly the same silencing (54.97%), comparable to the targeted siPCSK9@NPs‐aL group. Western blot analysis was performed in oxLDL‐pretreated HUVECs (Figure S5). Treatment with siPCSK9‐containing formulations significantly reduced PCSK9 protein expression while concomitantly increasing LDLR levels, consistent with restoration of LDLR recycling. Groups containing anti‐LOX‐1 conjugation exhibited marked downregulation of LOX‐1 protein. Importantly, none of the treatment groups affected CD36 membrane protein expression, highlighting the specificity of the PCSK9‐LOX‐1 targeting strategy. These protein‐level changes corroborate the qRT‐PCR results and provide direct evidence that siPCSK9@PEAL NPs‐aL simultaneously disrupts hepatic PCSK9‐driven hyperlipidemia and plaque‐localized LOX‐1‐mediated oxLDL uptake, offering a dual mechanism to interrupt the self‐amplifying axis in atherosclerosis.

Previous studies have established that oxLDL in the atherosclerotic inflammatory microenvironment significantly induces mitochondrial dysfunction, leading to mitochondrial metabolic reprogramming, amplified inflammation, and apoptosis [30, 31]. To determine whether siPCSK9@PEAL NPs‐aL mitigates oxLDL‐induced mitochondrial impairment, we assessed mitochondrial membrane potential (MMP) using JC‐1 staining in oxLDL‐pretreated HUVECs treated with different formulations (Figure 3E). In the NC group, oxLDL exposure caused marked MMP depolarization, as evidenced by a high green/red fluorescence ratio. The targeted siPCSK9@PEAL NPs‐aL group exhibited the most pronounced recovery, significantly lowering the green/red ratio compared to other groups.

To examine bioenergetic changes, we then performed Seahorse extracellular flux analysis to measure oxygen consumption rate (OCR) and extracellular acidification rate (ECAR) in oxLDL‐pretreated HUVECs after 24 h treatment (Figure 3F). PBS‐treated cells showed impaired mitochondrial function, with maximal respiration of 55.1 ± 1.4 pmol min^−1^ and spare respiratory capacity of 26.2 ± 0.9 pmol min^−1^. Notably, PEAL NPs‐aL alone significantly enhanced maximal respiration (70.9 ± 1.5 pmol min^−1^) and spare capacity (40.4 ± 0.8 pmol min^−1^), suggesting a direct benefit of LOX‐1 blockade on mitochondrial function. The siPCSK9@PEAL NPs‐aL group achieved the greatest restoration, with maximal respiration reaching 82.8 ± 1.9 pmol min^−1^ and spare capacity increasing to 45.2 ± 1.1 pmol min^−1^ (Figure 3G). For ECAR parameters, PBS‐treated cells exhibited elevated maximal glycolysis and glycolytic reserve, reflecting compensatory glycolytic shift due to mitochondrial impairment. The siPCSK9@PEAL NPs‐aL group showed substantial suppression of maximal glycolysis (65.8 ± 2.4 mpH min^−1^) and glycolytic reserve (8.6 ± 3.9 mpH min^−1^). PEAL NPs‐aL alone similarly lowered maximal glycolysis (66.1 ± 2.4 mpH min^−1^) and reserve (8.1 ± 2.1 mpH min^−1^) (Figure S6). These metabolic improvements demonstrate that siPCSK9@PEAL NPs‐aL restores mitochondrial respiratory capacity and attenuates compensatory glycolysis in oxLDL‐stressed endothelial cells. The strong effect of PEAL NPs‐aL alone suggests LOX‐1 blockade directly mitigates oxLDL‐triggered impairment, contributing to reduced oxidative stress and improved endothelial survival.

Take a step further, we measured apoptosis in oxLDL‐pretreated HUVECs using Annexin V/7‐AAD staining and flow cytometry (Figure 3G). The NC group exhibited high apoptotic percentage, while free siPCSK9 + aL and siPCSK9@PEAL NPs showed no significant reduction. PEAL NPs‐aL alone reduced apoptotic cells to 4.23%. The siPCSK9@PEAL NPs‐aL group provided the most potent protection, lowering apoptotic cells to 2.72%. These findings indicate that non‐targeted delivery fails to effectively rescue apoptosis in atherosclerotic endothelial cells, likely due to insufficient disruption of the PCSK9‐LOX‐1 axis. Colony formation assays were then performed in untreated and oxLDL‐pretreated HUVECs (Figure 3H). In untreated HUVECs, only free siPCSK9 + aL showed mild cytotoxicity, while all nanoparticle formulations exhibited no significant toxicity. In oxLDL‐pretreated HUVECs, the NC group formed few colonies. Free siPCSK9 + aL modestly improved 29% colony formation, siPCSK9@PEAL NPs enhanced it to ∼40%. Both PEAL NPs‐aL and siPCSK9@PEAL NPs‐aL groups achieved the highest proliferation recovery, with no significant difference between them. Above results indicate that dual targeting promotes endothelial repair by enhancing proliferation, potentially contributing to plaque stabilization. The comparable performance of PEAL NPs‐aL alone suggests that LOX‐1 blockade itself supports long‐term cell survival and recovery in the atherosclerotic microenvironment.

Collectively, these assays reveal that siPCSK9@PEAL NPs‐aL effectively ameliorates oxLDL‐induced mitochondrial dysfunction, metabolic imbalance, apoptosis, and proliferation impairment in endothelial cells. The superior therapy effect of the targeted formulation underscores the synergistic benefits of PCSK9‐LOX‐1 axis inhibition, providing a robust foundation for in vivo therapeutic evaluation.

### 2.5 Therapeutic Evaluation of Dual‐Targeting Nanotherapy in Advanced Atherosclerosis Mice

To assess the combinatorial anti‐atherosclerotic effects of PCSK9/LOX‐1 dual inhibition, 8‐week‐old male ApoE^−^/^−^ mice were fed a high‐fat diet for 30 weeks to establish advanced atherosclerosis [21, 22]. Treatment was initiated at week 31 with weekly tail‐vein injections and continued for 8 weeks (Figure 4A). Real‐time ultrasound monitoring of the abdominal aorta revealed time‐dependent therapeutic responses (Figure 4B). Compared with C57 control mice, PBS‐treated atherosclerotic mice showed progressive luminal narrowing with persistent hyperechoic signals, indicating uncontrolled plaque expansion. Free siPCSK9 + aL monotherapy exhibited only transient efficacy, with benefits diminishing by week 8. In contrast, nano‐formulated therapies improved outcomes: siPCSK9@PEAL NPs and PEAL NPs‐aL achieved 18.47% and 15.65% plaque suppression at 12 weeks, respectively. The dual‐targeted siPCSK9@PEAL NPs‐aL group maintained luminal integrity throughout the observation period, reducing intimal hyperplasia by 32.09% compared to PBS.

**FIGURE 4:**
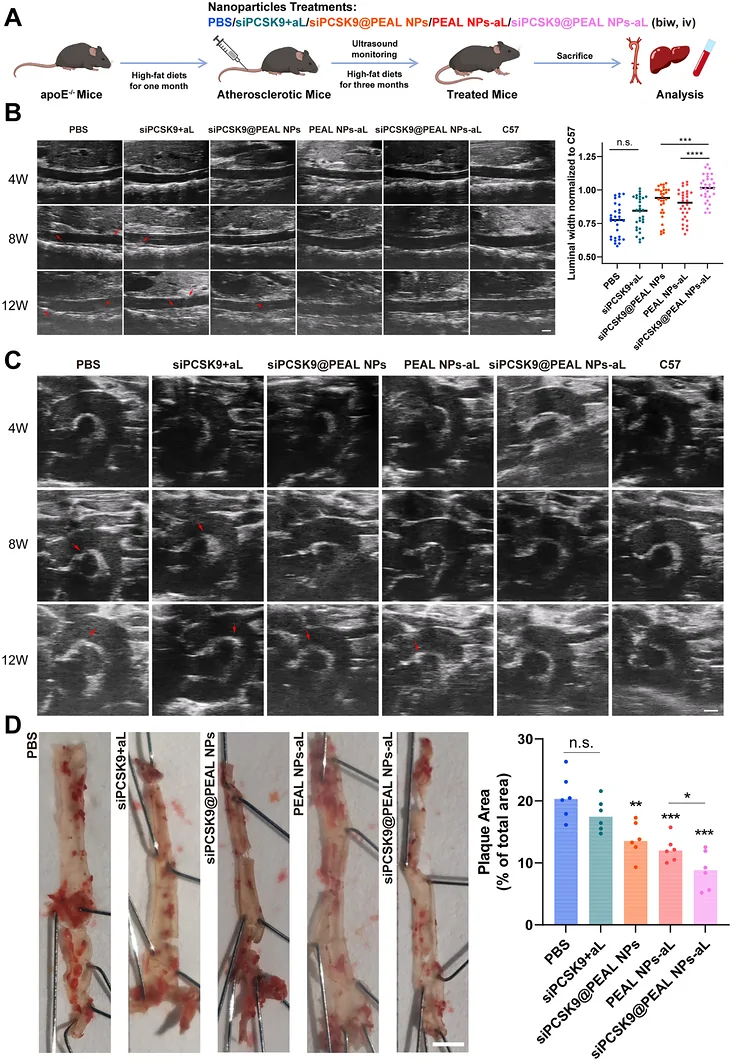
Administration of the dual‐targeted nanotherapy on atherosclerosis mice. (A) Schematic illustration of the atherosclerotic mice model construction and the anti‐atherosclerotic treatment protocol. (B) Representative ultrasound images of the abdominal aorta in B‐mode. Continuous strong echoes (white) along the lumen indicate plaque formation and narrowing. The atherosclerotic plaque is indicated by red arrows. Scale bar = 1 mm. The luminal width of the abdominal aorta at 12 weeks is quantified to evaluate the therapeutic effect. 5 representative widths form each image is calculated (*n* = 6). (C) Representative ultrasound images of the aortic arch in B‐mode. The atherosclerotic plaque is indicated by red arrows. Scale bar = 1 mm. (D) ORO‐stained aortas and quantitative analysis of the ORO‐positive areas in aortas with different treatments (*n* = 6). Scale bar = 2 mm. Data presented as mean ± SD with *p < 0.05, **p < 0.01, ***p < 0.001, ****p < 0.0001 and n.s. represents no significant difference.

Complementary ultrasound analysis of aortic arch lesions (Figure 4C) confirmed these findings. PBS‐treated mice developed confluent hyperechoic plaques, whereas dual‐targeted therapy significantly reduced lesion area. PCSK9 silencing alone moderately attenuated lipid accumulation, but its combination with LOX‐1 blockade synergistically stabilized advanced plaques. At the end of treatment, whole aortas were harvested and stained with Oil‐Red O (ORO) to quantify atherosclerotic plaques (Figure 4D). PBS‐treated controls showed the highest lipid deposition, while free siPCSK9 + aL reduced lipid area (not significant, p = 0.136). Single‐targeted therapies further decreased deposition: siPCSK9@PEAL NPs to 13.78 ± 2.87% and PEAL NPs‐aL to 12.20 ± 2.04%. The dual‐targeted siPCSK9@PEAL NPs‐aL group exhibited the lowest ORO‐positive area (8.82 ± 3.06%), consistent with ultrasound results and confirming superior efficacy of combined PCSK9 and LOX‐1 inhibition. These in vivo findings demonstrate that siPCSK9@PEAL NPs‐aL significantly suppresses plaque progression, lipid deposition, and intimal hyperplasia compared to monotherapy or single‐targeted approaches.

### 2.6 Targeting Inhibition of the PCSK9‐LOX‐1 Axis In Vivo

To elucidate the targeted inhibitory effects of dual‐targeted nanotherapy on the PCSK9‐LOX‐1 axis in vivo, we performed immunohistochemical and immunofluorescence analyses on aortic and hepatic tissues. Aortic sinus sections showed prominent LOX‐1 immunostaining in the PBS, siPCSK9 + aL, and siPCSK9@PEAL NPs groups, confirming sustained overexpression in atherosclerotic plaques (Figure 5A). In contrast, both PEAL NPs‐aL and siPCSK9@PEAL NPs‐aL groups exhibited markedly reduced LOX‐1 staining, demonstrating effective suppression of LOX‐1 at the plaque level through anti‐LOX‐1 antibody‐mediated targeting. Hepatic expression of PCSK9 and LDLR was assessed to evaluate systemic targeting (Figure 5B). PBS‐treated atherosclerotic mice displayed intense PCSK9 staining and weak LDLR expression, consistent with impaired LDLR recycling. PEAL NPs‐aL alone showed no effect on hepatic PCSK9/LDLR levels. Both siPCSK9@PEAL NPs and siPCSK9@PEAL NPs‐aL significantly reduced PCSK9 and restored LDLR expression. The dual‐targeted group achieved the most pronounced PCSK9 knockdown and LDLR upregulation, highlighting the benefit of combining plaque‐specific targeting with hepatic gene silencing.

**FIGURE 5:**
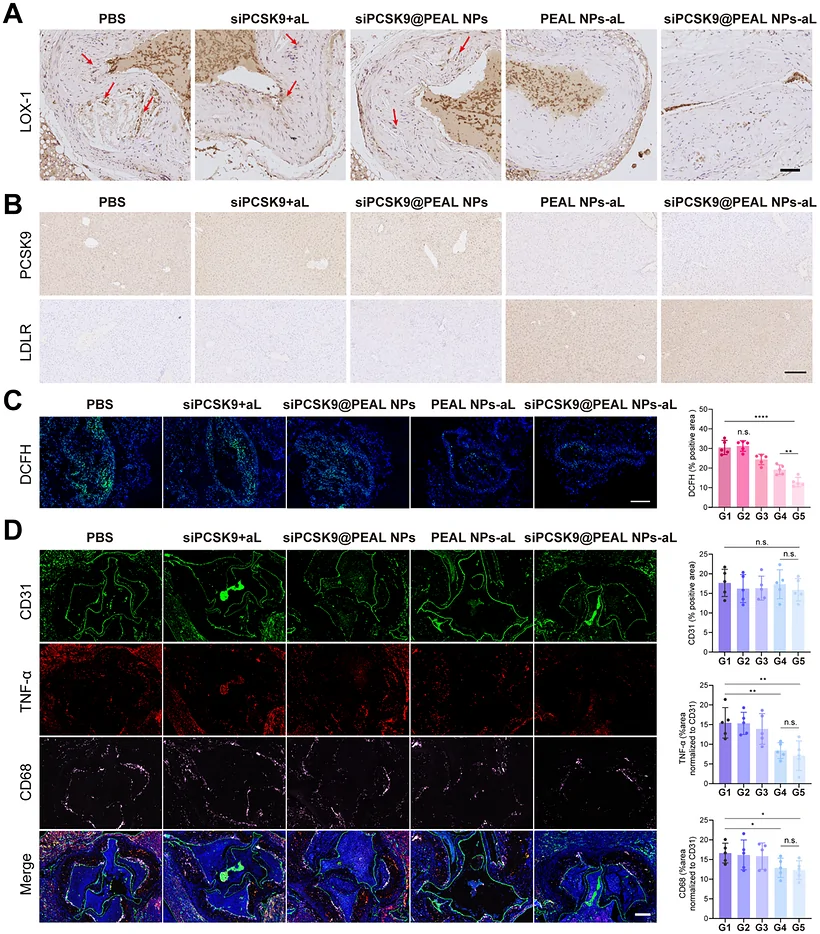
Targeted inhibition of dual‐therapeutic effects on target protein and inflammatory environment. (A) Representative images of aortic sinus sections stained with anti‐LOX‐1 antibody, highlighting significant stained areas (indicated by red arrows). Scale bar = 100 µm. (B) Representative images of liver tissue sections stained with PCSK9 and LDLR. Scale bar = 200 µm. (C) Confocal microscopy images of DCFH and DAPI‐stained aortic arches from atherosclerotic mice, accompanied by quantitative analysis of the relative DCFH fluorescence‐positive area. (D) Representative immunofluorescence micrographs of aortic root sections, with blue indicating nuclei (DAPI), red representing TNF‐α, and pink denoting CD68‐positive macrophages. Scale bar = 100 µm. Quantitative analysis of fluorescence‐positive areas is included. Groups: G1 (PBS), G2 (siPCSK9+aL), G3 (siPCSK9@PEAL NPs), G4 (PEAL NPs‐aL), G5 (siPCSK9@PEAL NPs‐aL). Data presented as mean ± SD with *p < 0.05, **p < 0.01, ***p < 0.001, ****p < 0.0001 and n.s. represents no significant difference.

Plaque oxidative stress and inflammation were further evaluated as downstream consequences of the PCSK9‐LOX‐1 axis [32, 33]. DCFH‐DA staining of aortic arches revealed intense fluorescence in PBS, siPCSK9 + aL, and siPCSK9@PEAL NPs groups (Figure 5C). Both PEAL NPs‐aL and siPCSK9@PEAL NPs‐aL significantly attenuated DCFH signal, with the dual‐targeted group showing the greatest reduction, indicating effective interruption of oxLDL‐driven ROS production via LOX‐1 blockade. Immunofluorescence co‐staining for TNF‐α and CD68‐positive macrophages in aortic root sections, with CD31 used to delineate vascular boundaries, revealed extensive macrophage infiltration and high TNF‐α expression within plaques in the PBS‐treated group (Figure 5D). Single‐modality treatments provided only modest improvement in reducing CD68+ area and TNF‐α intensity. In contrast, PEAL NPs‐aL and especially siPCSK9@PEAL NPs‐aL markedly decreased both CD68+ macrophage infiltration and TNF‐α expression within the CD31‐defined vascular regions. Quantitative analysis confirmed the most significant suppression of inflammatory markers in the dual‐targeted group, highlighting its superior ability to mitigate plaque inflammation by simultaneously disrupting systemic LDL formation and local oxLDL uptake.

These results collectively demonstrate that siPCSK9@PEAL NPs‐aL precisely inhibits both arms of the PCSK9‐LOX‐1 axis in vivo: hepatic PCSK9 silencing restores LDLR and reduces circulating LDL‐C substrate for oxLDL formation, while plaque‐targeting LOX‐1 blockade directly suppresses local oxLDL uptake, oxidative stress, and inflammation.

### 2.7 Systemic Therapeutic Evaluation of Dual‐Targeting Nanotherapy

Histopathological assessment of aortic sinus sections provided key validation of therapeutic specificity (Figure 6A and Figure S7). H&E staining revealed severe atherosclerotic burden in PBS‐treated controls, consistent with ultrasound and en face ORO findings. Free siPCSK9 + aL failed to reduce plaque progression. Single‐targeted nano‐formulations showed partial efficacy: siPCSK9@PEAL NPs reduced lesion area to 25.23 ± 3.97% and PEAL NPs‐aL to 25.45 ± 3.56%. Dual‐targeted siPCSK9@PEAL NPs‐aL achieved the most significant plaque reduction (20.72 ± 2.62%), confirming consistent therapeutic effects across aortic subregions. Masson's trichrome staining highlighted collagen deposition as a marker of fibrosis [34, 35] (Figure 6A). PBS‐treated mice displayed extensive blue collagen staining, whereas the dual‐targeted group showed noticeable reduction in collagen area, qualitatively indicating decreased fibrosis. α‐SMA staining for smooth muscle cells [36] revealed increased brown staining in both PEAL NPs‐aL and siPCSK9@PEAL NPs‐aL groups, with the dual‐targeted treatment exhibiting the most pronounced area, suggesting enhanced smooth muscle content and improved plaque stability (Figure 6A). ORO staining of aortic sinus sections further demonstrated reduced lipid deposition in nano‐formulated groups, with the lowest lipid accumulation in the siPCSK9@PEAL NPs‐aL group (Figure 6A).

**FIGURE 6:**
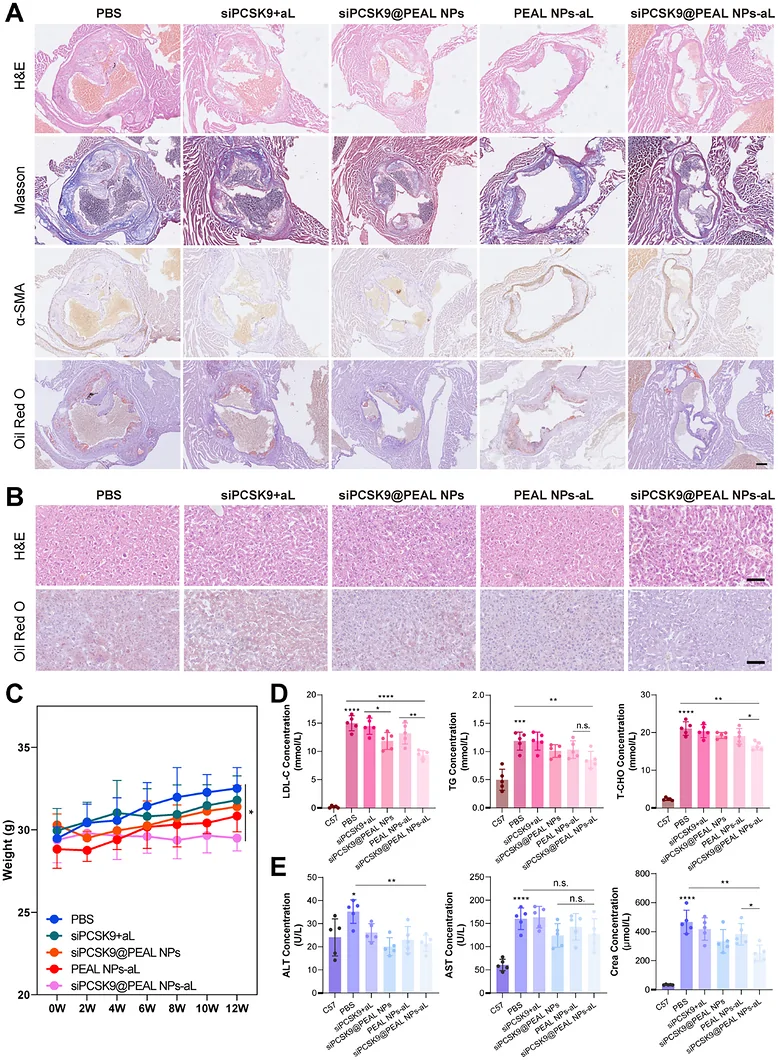
Evaluation of the dual‐targeted therapeutic effect on targeted organs. (A) Representative images of aortic sinus sections stained with H&E, Masson, α‐SMA, and ORO. Scale bar = 200 µm. (B) Representative images of liver tissue section stained with H&E and ORO. Scale bar = 200 µm. (C) Body weight of mice with time under various treatments. (D) Serum levels of TG, LDL‐C, and T‐CHO in all groups (*n* = 5). (E) Serum levels of ALT, AST, and Crea in all groups (*n* = 5). Data presented as mean ± SD with *p < 0.05, **p < 0.01, ***p < 0.001, ****p < 0.0001 and n.s. represents no significant difference.

Liver sections were examined to evaluate the impact of dual‐targeted therapy on hepatic lipid metabolism (Figure 6B). H&E staining showed severe edema in the atherosclerotic model [37]. Treatment with siPCSK9@PEAL NPs‐aL restored relatively normal cell morphology with only mild intercellular gaps, suggesting effective mitigation of atherosclerosis‐induced liver edema through modulation of lipid‐related pathways. ORO staining revealed reduced lipid deposition in nano‐formulated groups, particularly siPCSK9@PEAL NPs‐aL, compared to PBS and free siPCSK9 + aL controls, further supporting alleviation of hepatic lipid burden.

Body weight monitoring over the treatment period (Figure 6C) showed progressive increase in the atherosclerotic model due to high‐fat diet and lipid dysregulation. The dual‐targeted siPCSK9@PEAL NPs‐aL group exhibited a significant reduction in body weight compared to PBS, indicating improved fat metabolism Serum lipid profiles were analyzed at the end of treatment (Figure 6D). PBS‐treated mice displayed markedly elevated LDL‐C, siPCSK9‐loaded nanotherapies significantly lowered LDL‐C, while PEAL NPs‐aL alone showed only modest reduction. TG and total cholesterol (T‐CHO) levels followed a similar downward trend in the dual‐targeted group, reinforcing effective regulation of lipid metabolism via hepatic PCSK9 silencing. HDL‐C remained unchanged across treatments (Figure S8A), indicating selective modulation of atherogenic lipids.

Liver and kidney function markers were assessed for systemic safety (Figure 6E). As anticipated, atherosclerotic mice exhibited significantly elevated ALT and AST levels compared with C57BL/6J controls. siPCSK9@PEAL NPs‐aL treatment significantly normalized ALT, while AST remained insignificantly decreased across groups, indicating the dual‐targeting therapy has limited benefits on AST burden. Creatinine (Crea) levels improved significantly after dual‐targeted therapy, whereas Urea showed no change (Figure S8B). Importantly, no treatment group displayed further elevations in liver or kidney function markers beyond those induced by the disease model itself.

These results collectively demonstrate the favorable biocompatibility of siPCSK9@PEAL NPs‐aL. However, we acknowledge that the current study primarily focused on short‐term safety. Long‐term biocompatibility and potential immunogenicity will require further investigation with extended observation periods and toxicological assessments. Overall, these systemic evaluations confirm that dual‐targeted nanotherapy effectively reduces hepatic lipid burden, improves serum lipid profiles, and maintains organ function, highlighting its comprehensive therapeutic benefit and safety in advanced atherosclerosis.

### 2.8 Precise Modulation of Plaque Lipidome Revealed by Lipidomics Analysis

To further elucidate the precise impact of dual‐targeted nanotherapy on local lipid reprogramming at the molecular level, we performed non‐targeted lipidomics analysis on oxLDL‐pretreated HUVECs following different treatments. Based on the PLS‐DA model (Figure S9), a total of 926 differential lipids were identified using VIP > 1 and p < 0.05 criteria, with 314 lipids upregulated and 612 downregulated in the treatment group (siPCSK9@PEAL NPs‐aL) compared to PBS controls. Clustering heatmap (Figure 7A) and volcano plot (Figure 7C) clearly distinguished expression patterns between the control and treatment groups. Key lipids associated with oxLDL (highlighted in red rectangles) showed significantly reduced abundance in the treatment group, indicating effective suppression of pro‐atherogenic lipid accumulation. Detailed differential lipids are listed in Table S1. The top 10 upregulated lipids included TG(P‐45:14_5:0), PI(18:0_19:3), and various CL and PC species, while the top 10 downregulated lipids were predominantly TG and PG species enriched in long‐chain polyunsaturated fatty acids (Table S1).

**FIGURE 7:**
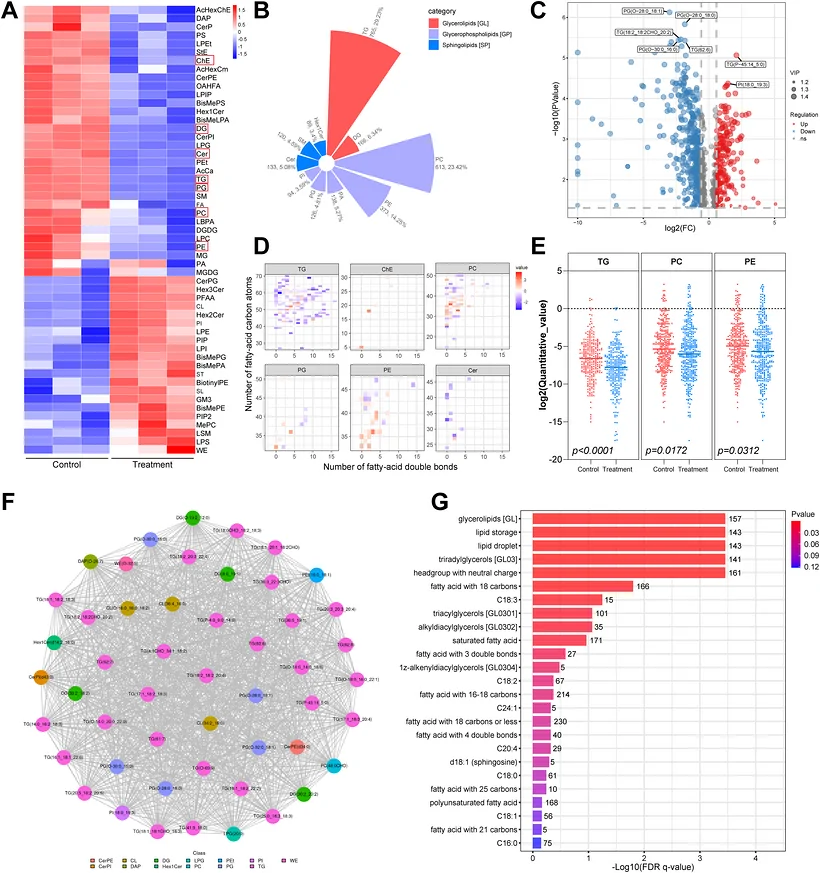
Evaluation of the dual‐targeted therapeutic effect by lipidomics. (A) The clustering heatmap illustrates the expression patterns of differential lipids between the Control and Treatment groups. Key lipids associated with oxLDL are highlighted with red rectangles for easy identification. (B) Pie chart illustrating the distribution of detected lipids, categorized by lipid functional roles. (C) The volcano plot depicts the distribution of differential lipids between the Control and Treatment groups, with the most significantly altered lipids specifically labeled to emphasize the impact of the treatment. (D) Structural heatmaps for each lipid class, including oxLDL‐related lipids: Each lipid species is represented by a rectangle, with color gradients indicating abundance differences between the Control and Treatment groups (red for increased abundance, blue for decreased abundance). Lipids are arranged by their degree of saturation (number of double bonds) along the x‐axis and by chain length (total carbon atoms) along the y‐axis. (E) Quantitative comparison of TG, PC, and PE lipid classes between the Control and Treatment groups, highlighting the treatment's effect on these lipid categories. (F) Spearman's correlation network (*p* < 0.05) depicting differential lipids in the Control vs. Treatment comparison. Circles, color‐coded by lipid categories, represent individual lipids. Connecting lines between nodes indicate correlation strength, with a higher number of lines signifying greater associations among differential lipids. (G) LION enrichment bar plot: The horizontal axis displays the −log10 FDR q‐value, while the vertical axis lists enriched entries from the LION database. Bar color and length represent the significance of the enrichment p‐value and the number of enriched lipids, respectively.

The pie chart (Figure 7B) summarized the distribution of detected lipids by functional category, with TG constituting the highest proportion (29.23%), followed by phosphatidylcholine (PC, 23.42%) and phosphatidylethanolamine (PE, 14.25%), highlighting the complex lipid composition in the atherosclerotic milieu. Structural heatmaps for major lipid classes (Figure 7D) were constructed by arranging lipid species according to degree of unsaturation (x‐axis: number of double bonds) and chain length (y‐axis: total carbon atoms), with color gradients indicating abundance changes (red: increased; blue: decreased) in the treatment group relative to control. The analysis revealed predominant downregulation of long‐chain, polyunsaturated oxLDL‐related lipids (particularly certain TG and PC species), while short‐chain or saturated lipids remained relatively stable, consistent with reduced oxidative stress and lipid peroxidation.

Quantitative comparison of major lipid classes (Figure 7E) confirmed that dual‐targeted treatment significantly lowered overall abundance of TG, PC, and PE (*p* < 0.01), aligning with the observed reductions in serum LDL‐C and plaque lipid deposition. Spearman correlation network analysis (*p* < 0.05) further illustrated associations among differential lipids (Figure 7F). Nodes were color‐coded by lipid category, with edge density reflecting correlation strength. In the treatment group, networks involving oxLDL‐related lipids became markedly sparser, indicating disruption of synergistic regulatory interactions among pro‐atherogenic lipids. Lipid Ontology (LION) enrichment analysis was performed on the downregulated lipid set identified in the treatment group (Figure 7G). The analysis revealed significant enrichment (−log10 FDR q‐value) in several biologically relevant categories, with the top‐ranked terms including glycerolipids, lipid storage, lipid droplet, triradylglycerols, and headgroups with neutral charge. These enriched terms are closely associated with storage lipids (particularly triacylglycerols), lipid droplet formation, and neutral lipid metabolism—key processes implicated in oxLDL‐driven lipid accumulation, foam cell formation, and plaque progression in atherosclerosis.

Collectively, these lipidomics results demonstrate that siPCSK9@PEAL NPs‐aL precisely reprograms the plaque lipidome by selectively downregulating oxLDL‐related pro‐atherogenic lipids, disrupting their correlative networks, and enriching for protective lipid ontology terms. This molecular‐level modulation aligns with reduced oxidative stress, inflammation, and lipid deposition observed in vivo, providing mechanistic evidence for the dual‐targeted strategy's efficacy in interrupting the PCSK9‐LOX‐1 axis‐driven lipid pathology in atherosclerosis.

## 3 Conclusions

Dual‐targeted nanotherapeutic system capable of achieving plaque‐hepatic targeting and intervening in multiple stages of disease progression may open new opportunities for managing complex systemic‐local diseases. Given the multifactorial pathological processes’ nature of atherosclerosis, effective intervention requires not only simultaneous suppression of different stages of oxLDL metabolism but also precise targeting to modulate inflammation, oxidative stress, and intracellular metabolism. In this study, we developed a dual‐targeted nanoparticle siPCSK9@PEAL NPs‐aL as a novel strategy. By integrating siRNA‐mediated PCSK9 silencing and anti‐LOX‐1 monoclonal antibody in a single nanoplatform, this system simultaneously inhibits PCSK9‐driven oxLDL generation and LOX‐1‐mediated oxLDL uptake, effectively disrupting the self‐amplifying PCSK9‐LOX‐1 positive feedback axis in atherosclerosis. This approach addresses a critical limitation of current PCSK9/LOX‐1‐targeted monotherapies, which fail to simultaneously control systemic oxLDL levels and local plaque inflammation [17, 38]. The design employs optimized particle size to enable passive hepatic targeting while minimizing rapid clearance by the RES, thereby achieving efficient siRNA delivery to hepatocytes. Meanwhile, surface functionalization with anti‐LOX‐1 mAb enables active plaque targeting to LOX‐1‐overexpressing endothelial cells. In vitro, siPCSK9@PEAL NPs‐aL effectively silenced PCSK9, downregulated LOX‐1 expression, rescued mitochondrial function, inhibited compensatory glycolysis, reduced cell apoptosis, and promoted proliferation. In ApoE^−^/^−^ mice with advanced atherosclerosis, weekly intravenous administration significantly reduced aortic plaque burden, attenuated fibrosis, stabilized plaque composition, and normalized serum LDL‐C, TG, and T‐CHO levels while alleviating hepatic lipid accumulation. More broadly, this approach addresses the clinical limitation of current PCSK9‐targeted therapies, which fail to adequately control local plaque inflammation and oxidative stress [10, 39]. By overcoming the low targeting efficiency and rapid RES clearance commonly associated with conventional nanocarriers, this spatially controlled, synergistic strategy provides a solid foundation for future drug development in atherosclerosis [40]. Looking ahead, such plaque‐hepatic dual‐targeting nanoplatforms hold promises for extension to other systemic‐local diseases, offering conceptual validation for next‐generation precision interventions and advancing the clinical translation of advanced biomaterials for atherosclerosis therapy.

## 4 Methods

### 4.1 Materials, Cells, and Animals

Anti‐LOX‐1 monoclonal antibody was purchased from Abcam (catalog no. EPR20750). Anti‐PCSK9 antibody was purchased from Sino Biological (Cat: 50251‐T24). Anti‐LDLR antibody was purchased form NOVUS (NBP2‐80833). Recombinant human PCSK9 (rhPCSK9) was obtained from Sino Biological. siPCSK9 and scramble control siRNA were synthesized and purified by Sangon Biotech Company using reversed‐phase high‐performance liquid chromatography (HPLC). AF488‐labeled siRNA and mNeonGreen were custom‐synthesized by Sangon Biotech. EDC (1‐ethyl‐3‐(3‐dimethylaminopropyl)carbodiimide) and NHS (N‐hydroxysuccinimide) were obtained from Sigma‐Aldrich. RNase A was purchased from Thermo Fisher Scientific. OxLDL was purchased from Yiyuan Biotechnologies. Oil‐Red O staining kit, JC‐1 mitochondrial membrane potential assay kit, DCFH‐DA ROS probe, and Annexin V/7‐AAD apoptosis detection kit were obtained from Beyotime Biotechnology. Seahorse XF Cell Mito Stress Test Kit and Glycolysis Stress Test Kit were purchased from Agilent Technologies. All other common reagents were of analytical grade and obtained from Sigma‐Aldrich or local suppliers.

Human umbilical vein endothelial cells (HUVECs) were purchased from American type culture collection (ATCC) and cultured in DMEM supplemented with 5% FBS (Gibco), and penicillin‐streptomycin (P/S, 100 IU mL^−1^, Gibco) at 37°C in a humidified incubator containing 5% CO_2_. AML‐12 mouse hepatocytes and RAW264.7 mouse macrophages were obtained from ATCC and maintained in specific medium under the same conditions.

Male C57BL/6J mice and ApoE^−^/^−^ mice, aged 8 weeks at the start of experiments, were purchased from GemPharmatech. All animal experiments were conducted in accordance with protocols approved by the Institutional Animal Care and Use Committee (IACUC) of Renji Hospital, approval number (License no. SYXK (Hu) 2017–0011). Mice were housed under specific pathogen‐free conditions with a 12 h light/dark cycle and free access to food and water. High‐fat diet (HFD, 45% kcal from fat) was used to induce advanced atherosclerosis in ApoE^−^/^−^ mice for 30 weeks. Animals were sacrificed by CO_2_ asphyxiation when experimental endpoints were reached or when humane endpoints were observed.

### 4.2 Synthesis of siPCSK9@PEAL NPs‐aL

The PEG‐PLGA‐PLL triblock copolymer (PEG molecular weight 5 kDa, PLGA 50:50 lactic:glycolic acid ratio, molecular weight 10 kDa, PLL molecular weight 5 kDa; Polyscitech) was carboxylated to provide reactive sites for antibody conjugation. Briefly, PEG‐PLGA‐PLL (100 mg) was dissolved in 5 mL anhydrous dichloromethane (DCM). Succinic anhydride (20 mg, 0.2 mmol) and triethylamine (TEA, 50 µL) were added, and the mixture was stirred at room temperature for 12 h. The carboxylated polymer was precipitated in cold diethyl ether (20 mL), collected by centrifugation (5000 rpm, 10 min), washed twice with diethyl ether, and dried under vacuum. Carboxylation was confirmed by FTIR spectroscopy, showing a new C = O peak at ∼1628 cm^−1^.

Anti‐LOX‐1 monoclonal antibody (aL, 1 mg mL^−1^ in PBS, pH 7.4) was covalently conjugated to the carboxylated polymer using EDC/NHS chemistry. Carboxylated PEG‐PLGA‐PLL (10 mg) was dissolved in 2 mL MES buffer (0.1 M, pH 6.0). EDC (2 mg, 10.4 µmol) and NHS (1.2 mg, 10.4 µmol) were added and stirred for 15 min at room temperature to activate carboxyl groups. The activated polymer solution was then added dropwise to the anti‐LOX‐1 antibody solution (0.5 mg in 1 mL PBS, pH 7.4), and the mixture was stirred gently at 4°C for 4 h. Conjugation efficiency was confirmed by SDS‐PAGE with Coomassie staining.

siPCSK9‐loaded nanoparticles were prepared using a double‐emulsion solvent evaporation method combined with electrostatic adsorption. Briefly, siPCSK9 (100 µg in 100 µL RNase‐free water) was added to a solution of antibody‐conjugated PEG‐PLGA‐PLL (10 mg in 1 mL DCM) at the optimized N/P ratio of 10:1. The mixture was sonicated (50 W, 30 s on/10 s off, 3 cycles) to form the primary W/O emulsion. This was then added to 5 mL PVA solution (1% w/v in water) and sonicated again (100 W, 1 min) to form the W/O/W emulsion. The solvent was removed by dialysis in PBS overnight. Nanoparticles were collected by centrifugation (12 000 rpm, 20 min, 4°C), washed with RNase‐free water, and resuspended in PBS. siRNA encapsulation efficiency was determined by RiboGreen kit (ThermoFisher, R11490), and particle size was confirmed by DLS using a Malvern Zetasizer Nano ZS90 (Malvern, U.K.). The final siPCSK9@PEAL NPs‐aL formulation was lyophilized with 5% mannitol as cryoprotectant and stored at −80°C until use. All steps involving siRNA were performed under RNase‐free conditions to prevent degradation.

### 4.3 SiRNA Encapsulation Efficiency and RNase Protection Assay

siRNA encapsulation efficiency (EE%) was determined using the RiboGreen assay (ThermoFisher, R11490). Nanoparticles were centrifuged (12 000 rpm, 20 min, 4°C), and free siRNA in the supernatant was quantified. EE% was calculated as [(total siRNA – free siRNA)/total siRNA] × 100%. For the RNase A protection assay, free siPCSK9 and siPCSK9@PEAL NPs‐aL (equivalent to 1 µg siRNA) were incubated with RNase A (0.1 µg mL^−1^) at 37°C for 5 min, 30 min, 1 h, 4 h, 12 h, 1 d, 2 d, and 3 d. Reactions were stopped by adding heparin (2 mg mL^−1^) to dissociate nanoparticles. Released siRNA was analyzed by 2% agarose gel electrophoresis (100 V, 30 min) and visualized under UV light using a Gel Doc system.

### 4.4 In Vitro pH‐Responsive Release Study

siRNA release from siPCSK9@PEAL NPs‐aL was evaluated using dialysis. Nanoparticles (equivalent to 10 µg siRNA) were placed in dialysis bags (MWCO 3.5 kDa) and dialyzed against 20 mL PBS at pH 7.4 or pH 6.0 (simulating plaque acidic microenvironment) at 37°C with gentle shaking. At predetermined time points (0.5, 1, 2, 4, 8, 12, 24, and 36 h), 1 mL release medium was withdrawn and replaced with fresh buffer. siRNA concentration was quantified by RiboGreen assay. Cumulative release (%) was calculated as (released siRNA/total loaded siRNA) × 100%.

### 4.5 Co‐Culture of Hepatocytes and Macrophages to Simulate Liver RES Microenvironment

AML‐12 mouse hepatocytes (2 × 10^5 cells/well) were seeded in the lower chamber of 6‐well Transwell plates with 3.0 µm pore size polycarbonate membranes (Corning). RAW264.7 mouse macrophages (1 × 10^6 cells/well) were seeded in the upper chamber overnight for stabilization. AF488‐labeled siRNA‐loaded PEAL NPs of different sizes (50, 100, 150, and 200 nm) were added to the upper chamber (macrophage side) and incubated for 24 h. Hepatocytes in the lower chamber were gently washed with PBS, detached using 0.25% trypsin‐EDTA, and collected for flow cytometry analysis. For cell‐type‐specific visualization by CLSM, hepatocytes or macrophages were grown on glass coverslips in the lower chamber.

### 4.6 Cellular Uptake Studies

mNeonGreen‐ or AF488‐siRNA‐loaded PEAL NPs (aL+ and aL‐) were incubated with oxLDL‐pretreated HUVECs (50–200 µg mL^−1^ NPs, 4–24 h). Uptake was assessed by confocal laser scanning microscopy (CLSM, Leica SP8) with DAPI nuclear counterstaining or by flow cytometry (BD FACSCanto II) using FITC channel. Data were analyzed with FlowJo software.

### 4.7 Gene Silencing and Protein Expression

oxLDL‐pretreated HUVECs were treated with free siPCSK9, siPCSK9@PEAL NPs, or siPCSK9@PEAL NPs‐aL (equivalent to 50 nM siRNA) for 48 h. Total RNA was extracted using TRIzol reagent and reverse‐transcribed with PrimeScript RT Master Mix (Takara). qRT‐PCR was performed using SYBR Green Master Mix on a QuantStudio 5 system (Thermo Fisher) with GAPDH as internal control. Relative expression was calculated by the 2^−ΔΔCt method. For protein analysis, cells were lysed in RIPA buffer with protease inhibitors. Proteins (30 µg/lane) were separated by SDS‐PAGE, transferred to PVDF membranes, and probed with primary antibodies against PCSK9, LDLR, LOX‐1, CD36, and β‐actin, followed by HRP‐conjugated secondary antibodies. Bands were visualized using ECL reagent and quantified with ImageJ.

### 4.8 Seahorse Extracellular Flux Analysis

OCR and ECAR were measured using the Seahorse XFe96 Analyzer (Agilent). Cells were seeded in XF96 plates (2×10^4 cells/well), pretreated with oxLDL, and treated for 24 h. After equilibration in XF base medium, sequential injections of oligomycin (1 µM), FCCP (1 µM), and rotenone/antimycin A (0.5 µM) were performed for OCR. For ECAR, glucose (10 mM), oligomycin (1 µM), and 2‐DG (50 mM) were injected. Data were normalized to cell number and analyzed using Wave software.

### 4.9 In Vivo Therapeutic Evaluation

ApoE^−^/^−^ mice were fed a high‐fat diet (45% kcal from fat) for 30 weeks to induce advanced atherosclerosis. Treatments were administered via tail‐vein injection weekly for 8 weeks starting at week 31. Abdominal aorta and aortic arch were monitored weekly by high‐resolution ultrasound (Vevo 2100, VisualSonics). At endpoint, mice were euthanized, and aortas were harvested for en face Oil‐Red O staining or aortic sinus cryosections for H&E, Masson's trichrome, α‐SMA, and ORO staining. Serum lipids (LDL‐C, HDL‐C, TG, T‐CHO) and liver/kidney function markers (ALT, AST, Crea, urea) were measured using commercial kits.

### 4.10 Lipidomics Analysis

Lipids were extracted from oxLDL‐pretreated HUVECs using the Folch method and analyzed by LC‐MS/MS (Waters ACQUITY UPLC coupled to Xevo G2‐XS QTOF). Data were processed with Progenesis QI software. Differential lipids were identified using VIP > 1 and *p* < 0.05 (PLS‐DA model). Heatmaps, volcano plots, structural heatmaps, Spearman correlation networks, and LION enrichment were generated using R packages (pheatmap, ggplot2, corrplot, LION).

### 4.11 Statistical Analysis

Data are presented as mean ± standard deviation (SD). Comparisons between groups were performed using one‐way ANOVA followed by Tukey's post hoc test or unpaired Student's t‐test. Statistical significance was set at *p* < 0.05 (*p < 0.05, **p < 0.01, ***p < 0.001, ****p < 0.0001). All analyses were conducted using GraphPad Prism 9.0.

## Author Contributions

Conceptualization, **Yi Duan**, **Yijie Qiu**, and **Yourong Duan**; methodology, **Yi Duan**, **Yijie Qiu**, **Quan Wang**, **Jiangtao Lin**, and **Yi Dong**; investigation, **Yan Zhu**, **Quan Wang**, **Qi Wang**, and **Jiangtao Lin**; visualization, **Yi Duan**, **Yijie Qiu**, **Yan Zhu**, **Yi Dong**, and **Yourong Duan**; supervision, **Yourong Duan**, **Yi Dong**, and **Qi Wang**; writing, **Yi Duan**, **Yijie Qiu**, **Yi Dong**, and **Qi Wang**; funding acquisition, **Yourong Duan** and **Yijie Qiu**.

## Conflicts of Interest

The authors declare no conflicts of interest.

## Supporting information




**Supporting File**: advs77100‐sup‐0001‐SuppMat.docx.

## Data Availability

The data that support the findings of this study are available from the corresponding author upon reasonable request.
