# Supplementary material for: Plaque‐Hepatic Targeting Nanotherapy Disrupts the PCSK9‐LOX‐1 Axis to Suppress oxLDL in Atherosclerosis
Source: Adv Sci (Weinh). 2026 Aug 13;13(61):e77100. doi: 10.1002/advs.77100 (PMC13472102; doi:10.1002/advs.77100)
Supplement: Supplementary file 1 [file ADVS-13-e77100-s001.docx]

**Supplementary Information**

**Plaque-Hepatic Targeting Nanotherapy Disrupts the PCSK9-LOX-1 Axis to Suppress oxLDL in Atherosclerosis**

Yi Duan^1, 2, #^, Yijie Qiu^1, #^, Yan Zhu^2^, Quan Wang^2^, Jiangtao Lin^2^, Yourong Duan^2, *^, Qi Wang^3, *^, Yi Dong^1, *^

*^1^*Department of Ultrasound, Xinhua Hospital, School of Medicine, Shanghai Jiao Tong University, 200092, Shanghai, China.

*^2^*State Key Laboratory of Systems Medicine for Cancer, Shanghai Cancer Institute, Renji Hospital, School of Medicine, Shanghai Jiao Tong University, Shanghai 200032, China.

*^3^*Key Laboratory for Advanced Materials and Institute of Fine Chemicals, Feringa Nobel Prize Scientist Joint Research Center, School of Chemistry and Molecular Engineering, East China University of Science and Technology, 200237, China.

^#^These authors contributed equally.

*Corresponding authors: Yi Dong; E-mail: drdaisydong@hotmail.com

Qi Wang; E-mail: wangqi@ecust.edu.cn

Yourong Duan; E-mail: yrduan@shsci.org

**Figure S1.** Western blot screening of key lipid metabolism-related proteins in aortic tissues from HFD-fed wild-type, ApoE⁻/⁻, and LDLR⁻/⁻ mice models of atherosclerosis.

**Figure S2.** Schematic of EDC/NHS-mediated covalent conjugation of anti-LOX-1 monoclonal antibody (aL) to carboxylated PEG-PLGA-PLL polymer.

**Figure S3.** Ex vivo fluorescence quantification results of the abdominal aorta at 2 h, 8 h, 24 h, and 48 h post tail-vein injection of free DiR or DiR@PEAL NPs-aL. Data are presented as mean ± SD. **p < 0.01, ***p < 0.001, (one-way ANOVA with Tukey’s post hoc test; n.s. = not significant).

**Figure S4.** Schematic diagram of the PCSK9-LOX-1 positive feedback axis in atherosclerosis and the mechanism of action of the dual-targeted nanotherapy. PCSK9 promotes LDLR degradation in hepatocytes, leading to increased circulating oxLDL. oxLDL binding to LOX-1 on endothelial cells activates pro-inflammatory and oxidative stress pathways, which in turn upregulates PCSK9 expression, forming a positive feedback loop. The siPCSK9@PEAL NPs-aL simultaneously achieves hepatic PCSK9 silencing and plaque-specific LOX-1 blockade, thereby disrupting this self-amplifying axis at both systemic and local levels.

**Figure S5.** Western blotting results of LDL-related key proteins after different treatments.

**Figure S6.** Maximal glycolysis and Glycolytic reserve quantification from Seahorse extracellular flux analysis (n=3). Data are presented as mean ± SD. *p < 0.05, **p < 0.01 (one-way ANOVA with Tukey’s post hoc test; n.s. = not significant).

**Figure S7.** Quantitative analysis of the relative plaque area in sections of the aortic sinus after H&E staining (n = 5). Data presented as mean ± SD with *p < 0.05, **P < 0.01, and n.s. represents no significant difference.

**Figure S8.** (A) Serum levels of HDL-C in all groups (n = 5). (B) Serum levels of Urea in all groups (n = 5). Data presented as mean ± SD with ****P < 0.0001 and n.s. represents no significant difference.

**Figure S9.** PCA score plot of the PLS-DA on lipidomics data of samples from the Treatment versus Control group.

**Table 1 Differential lipids identified in the dual-targeted nanotherapy versus PBS treated oxLDL-HUVECs**

Top 25 of up/down regulated lipids are shown, ranked by -log10(P. value).

| Accession | log2FC | -log10(P.value) | VIP | MainIon | mz | Regulation |
| --- | --- | --- | --- | --- | --- | --- |
| PG(O-28:0_18:1) | -3 | 6.13 | 1.47232 | M+NH4 | 920.76780 | Down |
| PG(O-28:0_18:0) | -1.84 | 5.83 | 1.47119 | M+NH4 | 922.78345 | Down |
| TG(18:2_18:2CHO_20:2) | -2.16 | 5.46 | 1.46961 | M+NH4 | 938.78073 | Down |
| PG(O-30:0_16:0) | -2.3 | 5.44 | 1.47115 | M+Na | 927.73884 | Down |
| TG(62:6) | -1.83 | 5.43 | 1.46960 | M+NH4 | 1008.89537 | Down |
| PC(48:0CHO) | -2.87 | 5.39 | 1.47165 | M+H | 972.79910 | Down |
| TG(20:3_20:3_20:4) | -2.96 | 5.31 | 1.46849 | M+NH4 | 972.80147 | Down |
| PG(O-30:0_15:0) | -2.08 | 5.29 | 1.47130 | M+NH4 | 908.76780 | Down |
| TG(16:1_18:1_22:6) | -4.12 | 5.14 | 1.46816 | M+NH4 | 920.77017 | Down |
| DG(O-19:2_12:0) | -17.03 | 5.14 | 1.46933 | M+Na | 559.46968 | Down |
| DG(30:2_20:2) | -1.77 | 5.06 | 1.46966 | M+NH4 | 830.75960 | Down |
| LPG(20:0) | -5.93 | 4.9 | 1.46780 | M+NH4 | 558.37655 | Down |
| TG(17:1_18:3_20:4) | -2.38 | 4.84 | 1.46817 | M+NH4 | 906.75452 | Down |
| TG(41:9_18:0) | -3.39 | 4.84 | 1.46967 | M+NH4 | 960.80147 | Down |
| TG(18:0CHO_18:2_18:3) | -1.88 | 4.83 | 1.47069 | M+NH4 | 912.76508 | Down |
| TG(25:0_18:3_18:3) | -1.63 | 4.76 | 1.46606 | M+NH4 | 994.87972 | Down |
| TG(18:2_20:3_22:4) | -2.39 | 4.71 | 1.46783 | M+NH4 | 974.81712 | Down |
| TG(18:1_18:1CHO_18:3) | -1.86 | 4.66 | 1.46882 | M+NH4 | 912.76508 | Down |
| TG(18:2_18:2_20:4) | -1.96 | 4.61 | 1.46603 | M+Na | 925.72556 | Down |
| TG(36:5_19:1) | -1 | 4.59 | 1.47058 | M+NH4 | 910.78582 | Down |
| TG(O-18:0_16:0_22:1) | -1.78 | 4.53 | 1.46828 | M+NH4 | 920.90045 | Down |
| TG(4:1CHO_34:1_18:2) | -1.87 | 4.52 | 1.46678 | M+NH4 | 942.81203 | Down |
| PG(O-32:0_18:1) | -1.77 | 4.51 | 1.46641 | M+NH4 | 976.83040 | Down |
| TG(61:7) | -2.08 | 4.48 | 1.46664 | M+NH4 | 992.86407 | Down |
| PEt(16:0_18:1) | -3.88 | 4.43 | 1.46623 | M-H | 701.51268 | Down |
| TG(P-45:14_5:0) | 2.09 | 5.07 | 1.47109 | M+H | 791.56090 | Up |
| PI(18:0_19:3) | 1.45 | 4.37 | 1.46824 | M-H | 873.54986 | Up |
| CL(O-16:0_16:0_18:2) | 1.39 | 4.32 | 1.46600 | M-H | 1137.73529 | Up |
| CL(34:2_16:0) | 1.31 | 4.31 | 1.46558 | M-H | 1137.73529 | Up |
| CL(36:4_16:0) | 1.23 | 4.28 | 1.46782 | M-H | 1161.73529 | Up |
| PC(16:0_24:0) | 2.01 | 4.13 | 1.46511 | M+H | 846.69463 | Up |
| BisMePE(47:1) | 1.48 | 4.1 | 1.46649 | M+Na | 950.75483 | Up |
| PC(32:1_18:2) | 1.25 | 3.82 | 1.46270 | M+HCOO | 1024.79511 | Up |
| PC(26:0_18:1) | 0.95 | 3.81 | 1.46271 | M+HCOO | 944.73251 | Up |
| PG(O-16:3_16:0) | 1.11 | 3.8 | 1.45755 | M+CH3COO | 761.49743 | Up |
| LBPA(14:0_18:2) | 1.5 | 3.78 | 1.46137 | M-H | 717.47121 | Up |
| MePC(38:1) | 1.25 | 3.74 | 1.45555 | M+NH4 | 848.69771 | Up |
| PG(O-26:5_20:4) | 0.49 | 3.71 | 1.45590 | M-H | 885.60150 | Up |
| PC(18:2_32:2) | 1.33 | 3.67 | 1.46134 | M+HCOO | 1022.77946 | Up |
| CL(32:0_18:1) | 1.21 | 3.67 | 1.45861 | M+H | 1141.76549 | Up |
| PC(24:0_18:2) | 0.8 | 3.64 | 1.45807 | M+HCOO | 914.68556 | Up |
| PC(42:0) | 3.03 | 3.63 | 1.45561 | M+H | 874.72593 | Up |
| CL(16:0_18:1_18:2) | 2.06 | 3.6 | 1.45991 | M-H | 1163.75094 | Up |
| MePC(41:0) | 2.82 | 3.6 | 1.45830 | M+H | 875.73376 | Up |
| TG(14:0_14:0_19:0COOH) | 2.54 | 3.59 | 1.44836 | M+NH4 | 840.72870 | Up |
| PC(20:0_17:1CHO) | 0.52 | 3.57 | 1.45703 | M-H | 814.59675 | Up |
| PC(30:0) | 1.36 | 3.53 | 1.45135 | M+Na | 728.52008 | Up |
| PFAA(28:6) | 1.72 | 3.47 | 1.45473 | M+Na | 434.33934 | Up |
| Hex2Cer(d18:1_18:0) | 0.69 | 3.47 | 1.45359 | M+HCOO | 934.64725 | Up |
| PE(33:0) | 0.82 | 3.45 | 1.45591 | M+Na | 728.52008 | Up |
